# Program for education and enrichment of relational skills (PEERS) training for social skills and depressed mood intervention in young adult with depression: Study protocol for a randomized controlled trial

**DOI:** 10.3389/fpsyt.2022.993124

**Published:** 2022-09-12

**Authors:** Yuting Hua, Qiyuan Zhao, Jiantong Shen, Yujin Liu, Lei Zheng, Mei Zhang

**Affiliations:** Huzhou University, Huzhou, Zhejiang, China

**Keywords:** PEERS, youth depression, social function, depressed mood, randomized controlled trail

## Abstract

**Introduction:**

Depression is a common psychiatric disorder characterized by persistent low mood, reduced interest, and slowed thinking. Young adults are the main first-onset group for depression in all categories of the population. Program for education and enrichment of relational skills (PEERS) training, a program for the Education and Enrichment of Relational Skills, has been used in Europe and America for people with various types of social disorders with good results. A Chinese adaptation of the PEERS training program may be a new approach to help youth with depression return to society as soon as possible. This study aimed to construct and optimize a social skills training program for Chinese young adults with depression and to validate the impact of the program.

**Materials and methods and analysis:**

The aim of this trial protocol is to evaluate the efficacy of the localized PEERS training program on social competence, depressed mood in a Chinese young adult population with depression. The primary outcome will be a change in self-reported depressive symptoms from baseline to week 3 post-randomization to week 6 post-randomization measured using the Liebowitz social anxiety scale (LSAS). Secondary outcomes include the rate of decline in severe social anxiety, the Social Avoidance and Distress Scale (SAD), the Social Self-Efficacy Scale (PSSE), and the Hamilton Depression Scale (HAMD-17). Data for each assessment will be collected at baseline, week 3 of the trial, and week 6 of the trial.

**Ethics and dissemination:**

Ethics approval was obtained from the Hospital Ethics Committee. Findings will be disseminated through scientific journals, conferences, and university courses.

**Trial registration number:**

[http://www.chictr.org.cn/], identifier [ChiCTR2100046050].

## Introduction

Around the world, depression is one of the most common mental health problems and is ranked by the WHO as the single largest contributor to global functional disability (1). In fact, mental disorders account for 30% of the non-fatal disease burden worldwide and 10% of the overall disease burden, including death and disability (2). The total number of people living with depression worldwide in 2015 was estimated to be 322 million, or 4.4% of the total population. Nearly half of these people live in the South-East Asia Region, and Western Pacific Region (1), which include India and China, for example.

The prevalence of depression affects different age groups, and in various analyses of depression in youth, it was found that the prevalence of depression in young students was about 27.2% (3), about 5% of people aged 65 and over suffered from depression (4). In the first-episode group, there is a predominance of young adult (5). Youth morbidity can have a serious impact across the life cycle (6); the earlier the age of onset, the higher the risk of obsessive-compulsive, interpersonal sensitivity, depression, hostility, paranoid ideation, and psychoticism, the more pronounced the clinical features and the higher the degree of impaired social function (7). Early and effective intervention is therefore needed.

Research shows that people with depression experience impaired social functioning (6–9), even up to 20% of depressed people have severe social anxiety (9). The study finds depression with social anxiety associated with worsening depression (8, 10–12), cases with impaired social function have more physical symptoms, more residual symptoms of depression, and less satisfaction with the quality of life (13). After depressed disease are prone to heavy psychological burden and anxiety, fear and other negative emotions, but individual in society, the essential need to mix with all sorts of people, because of interpersonal communication is impaired, patients unable to integrate into the collective and society, unable to perform normal in daily work, and then form a vicious circle (14). While interpersonal harmony and emotional stability play complementary roles, social training can reduce social anxiety and reduce depressive symptoms. A study of prenatal depression patients who received 12-week social support training showed that attending a social support group reduced depression and social anxiety and improved intimacy (15). Through the vulnerability model of social skills deficit (16), Moeller RW verified that the low level of social skills of college students is correlated with the high rate of loneliness. The development of social skills programs can reduce the loneliness of college students with depression and anxiety, and further reduce their mental health burden (17). Early social skills training can be effective in reducing social anxiety and depression (18–20).

Clinical social skills training include interpersonal therapy (IPT) (21), social skills training (SST) (22), dynamic interpersonal therapy (DIT) (23), and so on. IPT is currently the mainstay of treatment for social dysfunction in patients with depression. It was originally developed by Gerald Klerman and Myrna Weissman as a psychological treatment for outpatients with severe depression (24), based on “psychobiological approach” of Adolf Meyers (25), “Interpersonal School” of Harry Stack Sullivans (26) and “Attachment theory” of John Bowlbys (27). Numerous clinical randomized controlled trials have confirmed the effectiveness of IPT in the treatment of patients with depression (28). But for now, IPT is more focused on patients with postpartum depression (21) and helping new mothers adjust to the role change. SST refers to helping patients simulate normal communication and communication methods by teaching patients’ social communication skills, so as to stimulate patients to find the joy of life and play a therapeutic purpose (22). The training includes four main areas: language skills, basic interpersonal skills, self-confidence, and skills in helping others and seeking help. DIT is based on building alliances of help and trust, and emphasizes the connection between this alliance and the patient’s past experiences (29). Take the lead of the therapist to help the patient relax and communicate effectively while dealing with interpersonal challenges (30). These social skills training exercises have been shown to be effective in guiding social function in people with depression, but they are mainly aimed at people of all ages with depression. The vast majority of evidence focuses only on depression itself, ignoring the differences in the context of the family environment at different ages, taking into account the age-specific nature of the youth population, which differs from adults and older people in the way they approach relationships (31). All of these training lack relevance to the youth depression population, and there is an urgent clinical need for a social intervention for a young adult with depression.

Program for Education and Enrichment of Relational Skills (PEERS) is a training program developed by Dr. Laugeson at UCLA (University of California, Los Angeles, Los Angeles, CA, United States) to address social skills deficits (32–34). It is a parent-assisted, manualized social skills training program for adolescents with ASD. PEERS training focus on key areas of social function for young people, including such as developing conversational skills, choosing appropriate friends, using humor, planning get-togethers, using good sportsmanship, and handling peer rejection, such as teasing physical bullying, gossiping, and disagreements. Teaching ecologically effective skills to nurture and maintain friendships using psycho-educational and cognitive-behavioral therapy techniques (35). PEERS training has been successively introduced and updated locally in Korea (36), Japan (37), Israel (38), the Netherlands (39), and Hong Kong (40), with good results in local ASD patients (41). Patients with ASD are often accompanied by depression, anxiety, intellectual disability, attention deficit, and other psychiatric comorbidities. The results of the study showed that after the PEERS intervention, patients’ social etiquette and social functioning scores improved significantly, their social communication skills, responsive and accepting behavior improved positively, and depressive symptoms were effectively reduced (40).

Since the development of PEERS training, a number of researchers have used various trials to evaluate the effect of PEERS training on the mental health of patients (42). It has been suggested that the PEERS program could help more people with impaired social functioning to exercise their social functions (40), such as people with depression. In North America, the PEERS training has been shown to be effective in reducing patients’ self-reported depressive symptoms (32) and has also been extended to patients with depression, anxiety, alcohol dependence, and other disorders (43, 44). This shows that PEERS training has a bright future as an intervention to improve social functioning, similar to IPT, SST, and DIT.

Considering the different cultural backgrounds, social civilizations, and interpersonal styles in mainland China, the applicability of the PEERS training to young Chinese patients with depression still needs to be further optimized and validated. Combining PEERS with the Chinese context and exploring the construction of a more suitable social training program for Chinese young people with depression has positive clinical implications for the new development of psychiatric treatment in China. Influenced by local, regional differences, this trial also provides a reference for the introduction of a locally adapted PEERS training program for depressed youth in countries around the world, contributing to the global spread of PEERS training programs.

### Objective

The aim of this protocol is to localize the PEERS training program in China, validate the efficacy of the Chinese PEERS training on social competence and depression in Chinese youth with depression, evaluate its effectiveness and safety during implementation so that it can better guide the establishment of clinical programs, provide evidence for the choice of new interventions. PEERS is a promising social intervention program that may be a useful addition to existing social intervention programs. The study will provide a reference for the introduction of locally adapted and targeted PEERS training, and promote the application of PEERS training program worldwide.

## Materials and methods and analysis

### Study design

**FIGURE 1:**
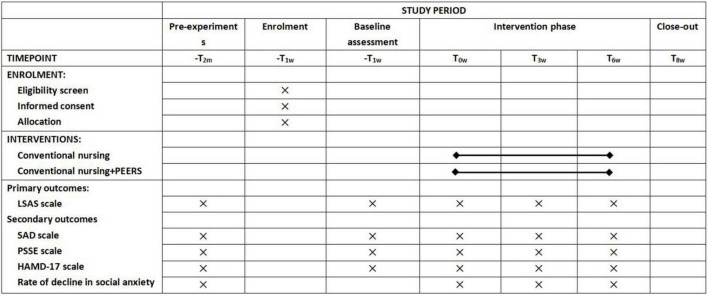
Schedule of enrollment, intervention, and assessment (m = month, w = week).

This study protocol conforms to the Standard Protocol Items: Recommendations for Interventional Trials (SPIRIT) guidelines (see Additional file 1) and accords with the SPIRIT Figure (Figure 1). The proposed PEERS training for the trial is based on literature research and expert discussion and is an optimized update of the original PEERS training program. Participant engagement in the trial will be divided into two phases, a non-intervention phase before randomization and an intervention phase 6 weeks after randomization. This trial was designed to validate the efficacy of the localized PEERS training, and therefore the control group in this trial will not receive any interventions other than usual care. Participants will be randomized by ward to receive the intervention, and due to the specific nature of the intervention, blinding will not apply to the interventionists, and the study will remain blind to scale assessors and data entry only.

### Participants

Trial participants were recruited from young adults with depression attending a specialist mental health hospital in Huzhou from July 2022 to January 2023. It is the largest specialist mental health hospital in the region, with psychological and psychiatric disorders diagnosis and treatment as its specialty, treating people with all types of psychosomatic disorders, with a wide range of influences and strong representation.

**FIGURE 2:**
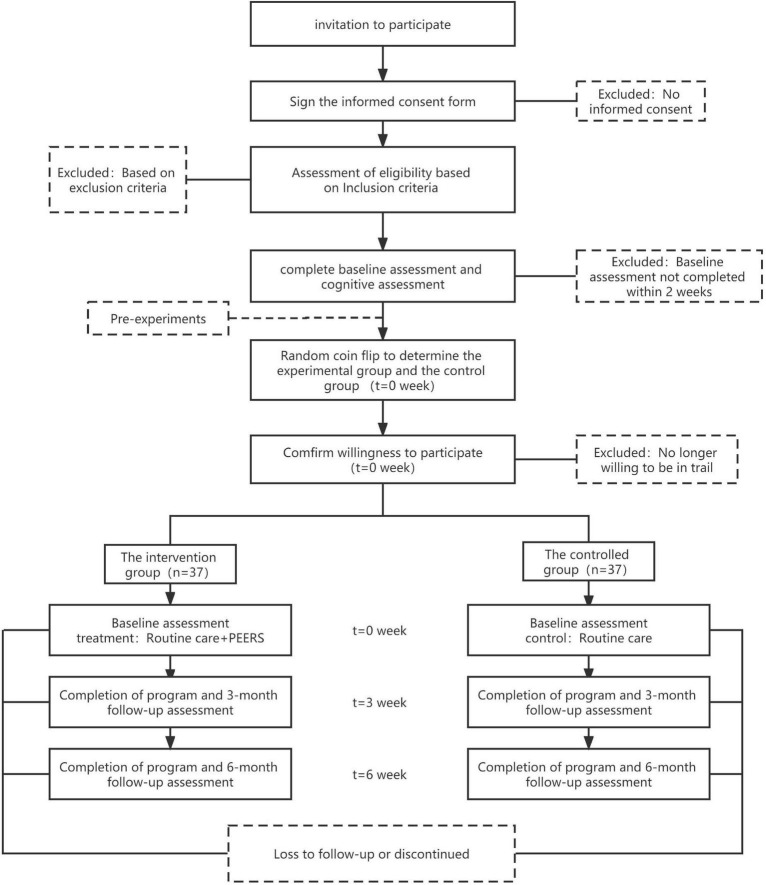
Participant timeline.

The purpose, procedures, possible risks, benefits, and other information about this trial will be explained in detail to participants. The trial participant schedule is specified in Figure 2.

### Inclusion criteria

① Aged 18–28°year (45).

② Willing and able to give informed consent.

③ The patient meets the diagnostic criteria for depression of the International Statistical Classification of Diseases and Related Health Problems 10th Revision (ICD-10) and gets HAMD-17 (≥ 18 points) and without other comorbid psychiatric disorders and two or more associate or chief physicians diagnosed with depression.

④ Patient has some degree of social impairment (LSAS ≥ 16 points).

⑤ Normal intelligence, without severe speech or emotional expression disorders.

⑥ Patients using SSRI antidepressants.

### Exclusion criteria

① Visual impairment, hearing impairment, or a serious physical illness that may affect the delivery of the intervention.
② Substance abuse and dependent patients.
③ Have a serious neurological disease or mental disorder.
④ Patients suffering from post-schizophrenic depression.
⑤ Bipolar disorder, depression in bipolar disorder.

### Withdrawal criteria

① Patient-initiated withdrawal from the trial.

② Non-cooperation of the patient during the trial.

③ Those whose condition worsened during the study and were transferred to an enclosed ward.

### Intervention

Both the intervention and control groups were given SSRI antidepressants and antidepressant psychotherapy. The control group was given conventional care, and the intervention group was given 6 weeks of PEERS intervention on top of conventional care. The intervention is delivered by a senior psychosomatic practitioner and a psychology teacher who provides verbal instructions, prompts, feedback, and assistance when needed.

The intervention will be carried out by members of the research team, who will be trained in relevant psychological knowledge and specific intervention methods, and will be assessed on their knowledge of depression and anxiety, the process of PEERS training, the details of implementation and precautions, etc.

### The controlled group

The control group will be given routine care based on normal administration of SSRI antidepressants and antidepressant psychotherapy (see Table 1 for details).

**TABLE 1:** Content of conventional nursing.

① Daily care	Assisting patients with daily care such as eating, hygiene, toileting, and sleeping
② Psychological care	For example, we understand in detail the causes of depression and related illnesses and take personalized care measures to guide patients to face illnesses and setbacks positively and happily.
③ Environmental care	Provide patients with a quiet and suitable care environment, keep the air in the room pleasant, open the windows for ventilation every day, practice gentle care, create suitable sleeping conditions for patients and keep them in a happy mood
④ Medication care	Observe drug efficacy and adverse effects when using SSRI antidepressants as prescribed by your doctor and provide timely feedback
⑤ Health education	Provide guidance on daily living skills, vocational skills, and social skills

### Intervention group

The intervention group implemented the PEERS training intervention (see Table 2 for details) on top of the normal administration of SSRI antidepressants and antidepressant psychotherapy and routine care, using different social themes to intervene with a fixed frequency for the patients, twice a week for 6 weeks. Each lesson uses a Socratic questioning approach to introduce questions to promote engagement with young depressed patients. Each lesson consists of four sessions, and the first session starts with feedback of the last assignment (8 min); the second session is a didactic presentation and teaching exercise led by the trainer (25–30 min); the third session is a rehearsal of the behavior by the participants themselves (10–20 min). The fourth session assigns socialization homework (2 min) to each patient, which is required to be completed at the end of the session to consolidate and improve on what has been learned in class.

**TABLE 2:** Course arrangement.

**Lesson**	**Intervention themes**	**Outline**	**Duration and frequency of intervention**
Lesson 1	Exchange of information and two-way communication to develop social networks	To communicate with each other by exploring the same interests and then forming a social network with like-minded people	Two lessons per week (one lesson per week in lesson 9/10), 45–60°min each
Lesson 2	Appropriate use of humor	Having a positive and optimistic mindset and learning the basic rules of appropriate humor	
Lesson 3	Small group strategy: Entry and exit	Assessing the acceptance of the small group you want to join, learn how to join the conversation with other young people and learn what to do when attempts fail	
Lesson 4	Interactive activities	How to organize and execute an in-house party and how to prepare, plan and execute an event well.	
Lesson 5	Good sporting spirit	How to perform well in video games, board games, sports, and other competitions	
Lesson 6	Handling ridicule	Counteracting through appropriate behavior to deal with unintentional verbal teasing from others	
Lesson 7	Dealing with bullying and changing a bad reputation	Includes long-term strategies for dealing with cyber violence, real-life bullying (e.g., physical threats) and changing a bad reputation	
Lesson 8	Resolving disagreements and arguments with friends	Specific steps to take when there is a disagreement or conflict with a friend	
Lesson 9	Dealing with rumors and gossip	Includes behavioral strategies to minimize the harm caused by gossip	
Lesson 10	Knowledge summary and review	A review of the social skills and knowledge learned and a graduation ceremony to round off the training.	

### Assignment of intervention

Prior to the start of the intervention, different ward were randomly divided into intervention and control groups using a coin toss by an investigator not involved in the implementation of the intervention to prevent contamination, and eligible subjects were sought within the respective ward.

### Outcome measure

The measurement of each scale measure, the outcome indicators will all be carried out by investigators who are not involved in the implementation of the intervention.

A baseline survey will be conducted by the investigator after the study subjects are identified, and basic information will be collected on the subjects, including age, gender, marital status, mode of payment (self-pay, agricultural insurance, medical insurance, and others), type of depression (general depressive disorder, anxiety depressive disorder, and others), previous history, family history, duration of illness and medication use, etc.

Considering that the first onset of action of antidepressants is between 2 and 4°weeks (46), We, therefore, chose to collect the subjects’ depression scores at three-time points: baseline, week 3 of the intervention and week 6 of the intervention. The scales were selected based on clinical applicability and reliability to dynamically assess the efficacy of the localized PEERS training.

### Primary outcome

Liebowitz social anxiety (LSAS) at week 3 and week 6 of the intervention will be assessed to measure social anxiety.

The LSAS scale was developed by Liebowitz in 1987 (47). It is commonly used to assess subjects’ social anxiety and is one of the most commonly used scales to provide a good picture of a subject’s social situation. The 24-item scale assesses fear in 11 social and 13 operational contexts. Scored on a four-point scale, for the fear subscale: 0 (none); 1 (mild), tolerable; 2 (moderate), distressing; 3 (severe), seriously interferes with daily life and work. For the avoidance subscale: 0 (never), 1 (occasionally), 2 (often), 3 (always). The final scores are added together, and the higher the total score, the more severe the social anxiety state. Mild anxiety is defined as a total score of less than 16, while severe anxiety is defined as a score of more than 60 (48). The Chinese version of the LSAS was tested for reliability and validity, and the Correlation between the items and the total score was good, the Cronbach’s α coefficient of each subscale was greater than 0.9, and the total scale score of the retest reliability was 0.779, the scale was generally satisfactory (49).

### Secondary outcome

① Rate of decline in severe social anxiety: A comparison will be made between the number of people in the intervention group and the control group with a decrease in severe anxiety as a percentage of the original number after week 6 to visually demonstrate the efficacy of PEERS training.
② Social avoidance and distress (SAD) scale: Compilated by WATSON et al. in 1969 (50), it contains 28 entries. The scale is a “yes-no” scale that assesses the tendency to avoid social interaction and subjective feelings of distress. The higher the patient’s score, the more severe the social avoidance and distress. The mean correlation index of the Chinese version of the SAD total score is greater than 0.75. The internal consistency reliability of the SAD total scale is greater than 0.85, and the avoidance and distress subscales are both above 0.72, which is generally satisfactory (51). Subjects will be administered questionnaires before the intervention, at week 3 of the intervention and at week 6 of the intervention to compare changes in social fear.
③ Social self-efficacy scale (PSSE) scale: Compilated by Smith and Betz in 2000 (52). The scale is a one-factor structured questionnaire developed for adults and university students, with 25 questions on a five-point scale from 1 to 5, with one being “not at all confident” and five being “completely confident.” The questionnaire covers six sections of social interaction, covering all aspects of social situations. The reliability coefficient of the Chinese version of the PSSE scale is above 0.92, with good construct validity; the Cronbach’s α coefficient is 0.90, indicating good internal consistency (53). Subjects will be administered questionnaires before the intervention, at week 3 of the intervention and at week 6 of the intervention to compare changes in social self-efficacy.
④ Hamilton depression scale (HAMD-17) scale: Developed by Hamilton in 1960, it is the most commonly used questionnaire for assessing depression in hospitals (54). Most of the HAMD-17 entries are scored on a five-point scale from 0 to 4. The scale is applied to adult patients with depressive symptoms, and the patient’s condition is assessed over a period of nearly one week. The total score is the sum of the factor scores; the higher the score, the more severe the condition. A total score of more than 24 may indicate severe depression, a score of more than 17 may indicate moderate depression, and a score of less than seven may indicate that the patient is not depressed. In a Chinese version of the HAMD-17 with 329 patients with depression, the inter-rater agreement met the criteria (Pan-kappa = 0.92), and the corresponding coefficient for internal consistency was 0.714, making the Chinese version of the scale very satisfactory (55). Subjects will be administered questionnaires before the intervention, at week 3 of the intervention and at week 6 of the intervention to compare changes in depressive symptoms.

### Sample size

#### Preliminary experiments

The purpose of the initial pre-experiment was to test the feasibility of the optimized PEERS training program in a young adult with depression. We included 12 young adults with depression, six in the intervention group and six in the control group. The intervention group used the PEERS to pre-experiment with the patients for a period of 6 weeks, following a lesson plan that closely followed the four components of feedback on assignments, didactic presentations, didactic exercises and structured training, and the control group had no intervention.

#### Official experiment

This trial is a clinical intervention study, and the primary outcome indicator is the LSAS score. Compare sample size formulae based on means $n=\frac{2^{2}\,{\left(\mu_{\alpha }+\mu_{\beta }\right)}^{2}\,\sigma^{2}}{\delta^{2}}$. Take α = 0.05, β = 0.10. From the pre-test, we can calculate that σ2 = 243.04 and δ2 = 272.25, estimating a sample size of about 62 cases, and considering a 20% missed visit, 62 + 62*0.2 = 74, 74 patients are to be included in the formal trial, 37 in the intervention group and 37 in the control group.

### Statistical analysis

#### Statistical data collection

The HAMD-17 scale will be administered by a professional psychologist, while the remaining LSAS, SAD, and PSSE scales will be completed on the spot by the assessor who will carefully explain the purpose of the study and the method of completion to the study participants and gain the support and understanding of the patients before completing the questionnaire. The scale assessors will be selected from those who do not perform clinical interventions to reduce bias due to subjective factors. A paper version of the questionnaire is given to inpatients, and patients are followed up by phone or text messages after they are discharged from the hospital. When all the scales have been collected, the information is promptly reviewed and confirmed, and patients are asked to cooperate in correcting any problems found.

Data entry will be carried out by investigators who are not involved in the implementation of the intervention. After the data has been safely collected and entered, the data collected will be statistically analyzed with SPSS 25.0. *P* < 0.05 means the differences are statistically significant.

### Statistical methods

For baseline comparisons, age, LSAS scores, SAD scores, PSSE scores, and total HAMD-17 scores are measurement data for both two groups and will be statistically described using the mean ± S.D., with Paired Sample *T*-Test used to assess between-group balance. The general profile of the test subjects, including gender, education level, occupation, marital status, mode of payment for medical care, per capita household income, type of depression, duration of illness, type of medication, family history, and past history, are all categorical data that will be statistically described using frequencies and percentages, and chi-square tests to assess between-group balance.

The primary outcome indicators (LSAS scores at week 3 and week 6 post-intervention for two groups of patients) and secondary outcome indicators (SAD, PSSE, HAMD-17 scores at week 3 and week 6 post-intervention for two groups of patients) will be compared using Repeated Measures ANOVA to analyze the efficacy of PEERS training at different time periods. Considering the possible interaction between intervention and time, we choose Simple Effects to analyze the different effects of different time variances on PEERS training.

### Patient and public involvement

Patients and/or the public were not involved in the design, or conduct, or reporting, or dissemination plans of this research.

### Ethics and dissemination

The trial has been approved by the hospital’s medical ethics committee and has been registered with the Chinese Clinical Trials Registry, ChiCTR2100046050, ID. Written consent will be sought from each participant prior to the formal trial, and no names or identifying information of participants will be disclosed. In addition, all participants have the right to withdraw from the trial at any time without consequence. The study will start in February 2022 and is expected to be completed in December 2022.

The findings of this study will be disseminated through scientific journals, academic conferences, and university courses.

## Discussion

The burden of illness caused by depressive disorders is high, both in terms of personal distress, impaired social functioning and relationships, reduced quality of life, and socio-economic costs (56). Evidence from numerous studies shows that people with depression suffer from a social impairment, which in severe cases affects their normal work life (9). It is vital to improve the current situation of impaired interpersonal barriers in people with depression and to develop their social skills and abilities. Several of the currently available interventions for social functioning have certain shortcomings of their own, in addition to being insufficiently targeted. For example, IPT is mostly individualized and requires one-to-one interaction between the therapist and the patient, which can consume more human, material and financial resources; SST contains fewer entries, and the training of the patient is not yet comprehensive and still needs to be enriched; DIT is quite demanding for the therapist, requiring him/her to calmly analyze the self, immerse himself/herself in psychoanalytic therapy and always bear in mind the exploratory and analytical nature of the method (57).

The PEERS program was initially developed as an empirically supported social skills intervention program for young people with autism (35), involving both parents and peers (58). In the subsequent development of PEERS, many scholars have verified that the PEERS program has a positive impact on the improvement of patients’ depressive mood (32, 59, 60). In terms of improving social functioning, the PEERS program has the unique advantage of adding the involvement of parents, peers and a greater need for peer socialization among the youth population, increasing the motivation of the youth depressed population to participate. As the most specific stage in the development of depression, young people need to deal with different social partners, and psychological interventions and practical training for depressed people during this period can yield better results (16, 17). PEERS is delivered as a course, which reduces human and material resources and allows for timely practice of what is learned in the course, with the trainer simply guiding and the classroom being more patient-centered, making it simple, effective, and easy to implement. The PEERS program meets the needs of depressed young people for peer socialization and fills the gaps in several existing social functioning intervention programs.

Compared with foreign cultures, Chinese people are more introverted in emotional expression, and Chinese young people with depression are more difficult to express their feelings. The PEERS program proposed for this trial combines the national conditions of the country and the physical and mental characteristics of young people with depression. Localized PEERS training program has been constructed by experts to make the content more scientific, purposeful and relevant based on access to a large database and expert working sessions. The optimized PEERS program compensates for the shortcomings of several existing social skills training programs mentioned above, observes the clinical manifestations and signs and symptoms of young depressed patients, combines their physical and psychological characteristics, simulates a real social environment, and trains depressed patients in small groups, enabling participants to enhance interactive communication.

The design of the experiment took into account the possibility of changes in the subject’s condition, and thus we set a 20% lost visit to ensure an adequate sample size. The intervention course is arranged in four interlocking sessions to refresh the knowledge taught and apply it to practice, while arranging for the coaches to take the lead and ensure the course is conducted properly, but more so to encourage the hands-on practice by the patients, using the class as an opportunity for patients to communicate and learn from each other, promoting their understanding of each other and helping each other to practice their social skills.

Before the formal trial began, a pre-experiment was conducted to examine both the motivation and cooperation of the study subjects. At the same time, the content of the sessions was improved to make the program more relevant to the actual needs of young people with depression, based on the subjects’ self-specific feelings and evaluations. The pre-experiment followed strictly to inclusion and exclusion criteria for the selection of subjects and was randomized to prevent cross-contamination between subjects. We found that all study participants cooperated positively with the study, were highly compliant and were able to successfully complete all training sessions without dangerous negative emotions or side effects, validating the reliability and safety of the training in this study. After the pre-experiment, social anxiety scores, social avoidance and distress mood decreased in the intervention group compared to the control group, while social self-efficacy scores increased relatively after the intervention, as detailed in Table 3.

**TABLE 3:** The grading of the pre-experiment (n1 = n2 = 6; LASA, Liebowitz social anxiety scale; SAD, social avoidance and distress scale; PSSE, scale of perceived social self-efficacy; HAMD-17, Hamilton depression scale).

Entries	Before the trial		After the trial	
	Intervention group	Control group	Intervention group	Control group
LSAS	55.50 ± 21.20	52.25 ± 20.30	41.75 ± 17.21	51.50 ± 13.90
SAD	22.50 ± 3.64	22.25 ± 3.90	16.25 ± 5.31	22.00 ± 4.30
PSSE	52.2 ± 10.35	43.75 ± 4.66	54.50 ± 7.92	45.00 ± 7.45
HAMD-17	19.25 ± 1.75	19.50 ± 1.19	10.75 ± 1.48	11.50 ± 2.06

If Chinese-specified PFEERS training is proven to improve depressed mood and enhance social skills in Chinese depressed youth populations, it could have significant implications for their recovery and prognosis. From a cross-diagnostic perspective, PEERS training may be a new time-saving and accessible method of training for health behavior change in other socially impaired populations, such as Chinese children with autism, youth with anxiety disorders, etc.

In conclusion, we hope that this study will provide evidence-based evidence for the application of PEERS training to the treatment of various types of socially impaired patients and provide a reference for the development of locally specific and targeted social skills training programs in each country.

## Ethics statement

The studies involving human participants were reviewed and approved by Huzhou Psychiatric Hospital’s Medical Ethics Committee. The patients/participants provided their written informed consent to participate in this study.

## Author contributions

YH, QZ, and JS conceived and designed the project. YH, QZ, JS, and YL oversaw data acquisition and data interpretation. YH, JS, QZ, and LZ performed the statistical analysis. YL and MZ checked the statistical analysis. YH and JS wrote the manuscript. All authors reviewed, revised, and approved the final version of the manuscript.
